# From Parent to Gamete: Vertical Transmission of *Symbiodinium* (Dinophyceae) ITS2 Sequence Assemblages in the Reef Building Coral *Montipora capitata*

**DOI:** 10.1371/journal.pone.0038440

**Published:** 2012-06-06

**Authors:** Jacqueline L. Padilla-Gamiño, Xavier Pochon, Christopher Bird, Gregory T. Concepcion, Ruth D. Gates

**Affiliations:** 1 Hawai’i Institute of Marine Biology, University of Hawai’i, Kaneohe, Hawai’i, United States of America; 2 Department of Oceanography, University of Hawai’i at Manoa, Honolulu, Hawai’i, United States of America; 3 Ecology, Evolution and Marine Biology, University of California Santa Barbara, Santa Barbara, California, United States of America; 4 Aquaculture and Biotechnology Division, Cawthron Institute, Nelson, New Zealand; 5 Cell Biology and Molecular Genetics, University of Maryland, College Park, Maryland, United States of America; Leibniz Center for Tropical Marine Ecology, Germany

## Abstract

Parental effects are ubiquitous in nature and in many organisms play a particularly critical role in the transfer of symbionts across generations; however, their influence and relative importance in the marine environment has rarely been considered. Coral reefs are biologically diverse and productive marine ecosystems, whose success is framed by symbiosis between reef-building corals and unicellular dinoflagellates in the genus *Symbiodinium*. Many corals produce aposymbiotic larvae that are infected by *Symbiodinium* from the environment (horizontal transmission), which allows for the acquisition of new endosymbionts (different from their parents) each generation. In the remaining species, *Symbiodinium* are transmitted directly from parent to offspring via eggs (vertical transmission), a mechanism that perpetuates the relationship between some or all of the *Symbiodinium* diversity found in the parent through multiple generations. Here we examine vertical transmission in the Hawaiian coral *Montipora capitata* by comparing the *Symbiodinium* ITS2 sequence assemblages in parent colonies and the eggs they produce. Parental effects on sequence assemblages in eggs are explored in the context of the coral genotype, colony morphology, and the environment of parent colonies. Our results indicate that ITS2 sequence assemblages in eggs are generally similar to their parents, and patterns in parental assemblages are different, and reflect environmental conditions, but not colony morphology or coral genotype. We conclude that eggs released by parent colonies during mass spawning events are seeded with different ITS2 sequence assemblages, which encompass phylogenetic variability that may have profound implications for the development, settlement and survival of coral offspring.

## Introduction

Parental effects are fundamentally important in biological systems. They occur when the phenotype of the offspring is affected by the phenotype or environment of the parents [1], [2]. These effects consequently influence the life history [3], competitive ability [4], evolutionary trajectories, speciation rates [5] and population dynamics [6], [7] of future generations of individuals. Parental effects can also mediate host pathogen relationships and facilitate the perpetuation of symbiosis between generations by influencing the direct transmission of symbiotic microorganisms from parent to progeny [8]. Although parental effects have been extensively studied in plants, insects and terrestrial vertebrates, these effects have received much less attention in the marine environment [1], [9].

In the ocean, a variety of algal and cyanobacterial symbionts live in association with protists and animal hosts in habitats ranging from the coastal sediments to the deep-sea hydrothermal vents [10]. Coral reefs are ecosystems whose ecological success is framed by endosymbiotic associations between scleractinian corals and unicellular dinoflagellates in the genus *Symbiodinium* [11]. *Symbiodinium* photosynthesis contributes to ecosystem productivity by translocating newly fixed carbon to its coral hosts, powering respiration and enhancing the deposition of calcium carbonate skeletons thereby creating a complex habitat for the extraordinary biodiversity characteristic of coral reefs [12], [13].

*Symbiodinium* is a taxonomically diverse genus comprising nine major lineages called clades A through I [14], that each contain from 2 - >100 subclade types [15]–[17]. Some coral species form highly specific associations with one or two closely related *Symbiodinium* types from one clade (e.g. Poritids, [16], [18]), while others form relationships with multiple *Symbiodinium* types that span the taxonomic breadth of the genus *Symbiodinium* (e.g. Pocilloporids, [18]). The taxonomic composition of *Symbiodinium* assemblages found at different locations on the same coral colony, within a single coral species sampled from different reefs, depths or different times of the year may also vary [15], [19], [20], illustrating a high degree of spatio-temporal variation in these endosymbiotic associations [21]–[23]. The broad taxonomic divisions in the genus *Symbiodinium* reflect in functional diversity, with distinct clades having different physiological optima [24]–[27]. Importantly, these differences in performance influence physiological characteristics of the coral host, rendering them more or less susceptible to environmental disturbances and disease (e.g. [15], [19], [22], [28]).

Given the fundamental role that *Symbiodinium* plays in the basic biology of corals, the perpetuation of this symbiosis is pivotal to the persistence of corals through time. Most coral species release asymbiotic eggs that are fertilized in the water column and which must acquire *Symbiodinium* from environmental pools (horizontal transmission) as planula-larvae and/or newly metamorphosed primary polyps [29], [30]. In other species, however, *Symbiodinium* are passed directly from the parent to the developing eggs (vertical transmission). These eggs are released (in mass or non-mass spawning episodes depending on the species), fertilized in the water column, and then develop into symbiotic larvae. For the species studied to date, approximately 25% of coral species that spawn gametes transmit *Symbiodinium* vertically through their eggs (n∼44 species) and 90% of corals that brood larvae (n∼36 species), release larvae containing *Symbiodinium* [31]. For example, the reefs of the Hawaiian Archipelago are dominated by coral species (from the genera *Montipora*, *Porites* and *Pocillopora*) that transmit *Symbiodinium* vertically [32]. In general, vertically transmitting species exhibit higher specificity in their endosymbiotic unions than horizontal transmitters [33]–[35]. *Symbiodinium* assemblages in coral offspring can be comprised of a single *Symbiodinium* type or a range of types, and as in adults, the taxonomic composition of these assemblages affects the growth and physiological tolerance of the juvenile coral [24], [25], [36].

No studies to date have examined the *Symbiodinium* assemblages in the eggs of a coral that transmits *Symbiodinium* vertically. For coral species that host diverse *Symbiodinium* assemblages, the transmission of *Symbiodinium* directly from parent to the egg potentially provides an opportunity for the parent to select the type(s) of *Symbiodinium* transmitted to the egg, and thus influence the physiological range, survival and recruitment success of their offspring. Further, differences in the endosymbiotic assemblages among adult colonies that reflect environmental conditions, life-history stage, health state or morphology may result in eggs seeded with very different *Symbiodinium* assemblages. Such parental effects have never been examined in corals but likely have implications for larval behaviors (swimming and habitat selection), natural selection after settlement, the potential for acclimatization to different environments and ultimately the resilience of coral populations and reef communities.

The timing of spawning in many corals is temporally constrained and predictable, making the collection and comparison of assemblages of *Symbiodinium* in newly spawned eggs tractable. We used this approach to examine the *Symbiodinium* ITS2 sequence assemblages (*sensu* [37]) transmitted from parents to eggs in the coral *Montipora capitata*, a simultaneous hermaphrodite that releases egg-sperm bundles during the new moon from late spring through summer in Hawai’i [38]. This coral is one of the most broadly distributed, morphologically plastic and important reef building corals in the main Hawaiian Islands [39], [40]. This combination of traits makes this species an excellent model for examining *Symbiodinium* transmission in the context of environment and colony morphology and genotype. Specifically, this study tests the hypotheses that (i) *Symbiodinium* ITS2 sequence assemblages between parent and eggs differ, and that (ii) differences in the ITS2 sequence assemblages in the eggs reflect differences in environmental conditions or morphology of the coral adult.

Studying vertical transmission of *Symbiodinium* can greatly enhance our understanding of how parental effects influence host-symbiont combinations in corals worldwide, which may play an important role in the physiological performance and stress tolerance of next generations and the future of coral reefs.

## Methods

### Ethics Statement

This study was conducted under the research guidelines of the University of Hawaii Executive Policy E5.211 and corals and gametes collected under the State of Hawaii Special Activity Permit numbers 2007-02, 2008-02 issued to the Hawaii Institute of Marine Biology.

### Study Sites and Sample Collections

**Figure 1:**
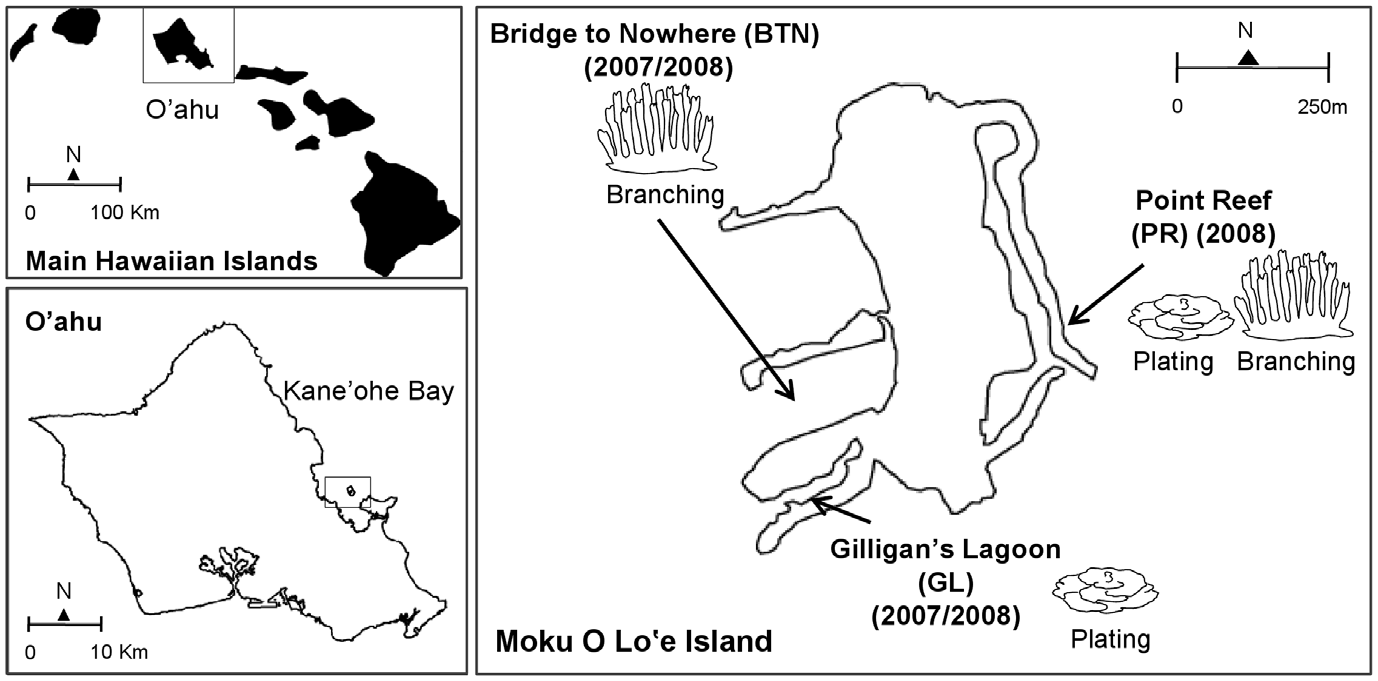
Location of study sites. Parent colonies and gametes were sampled at three sites around Moku O Lo’e Island: Bridge to Nowhere (BTN), Gilligan’s Lagoon (GL), and Point Reef (PR). Moku O Lo’e Island is located in Kane’ohe Bay on the windward coast of the island of O’ahu, Hawai’i, USA. *Montipora capitata* colonies exhibited primarily branching morphologies at BTN, plating at GL, and both branching or plating morphologies at PR.

**Figure 2:**
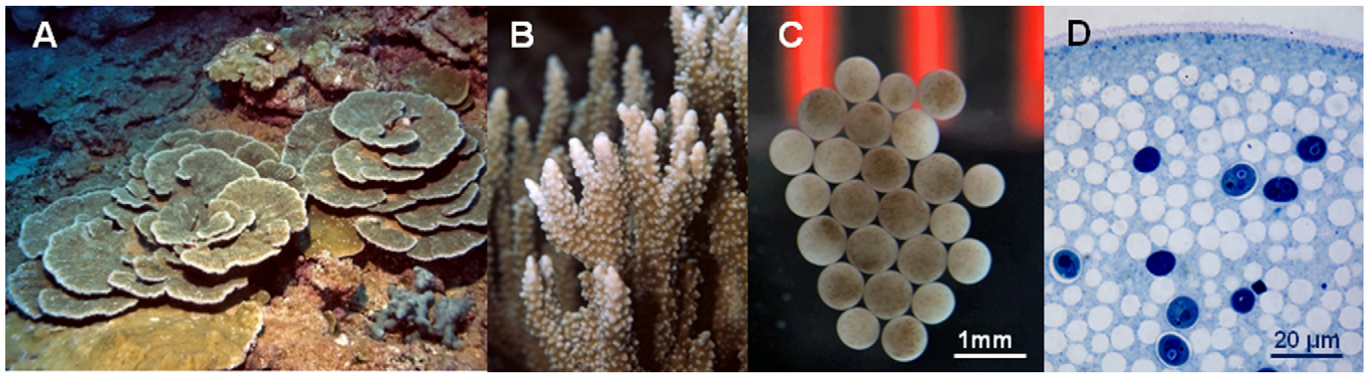
*Montipora capitata* colonies, their eggs and symbiotic *Symbiodinium* cells inside the eggs. (a) plating and (b) branching morphologies, (c) eggs seeded with *Symbiodinium* cells acquired from parent colonies and (d) close up of *Symbiodinium* cells inside the egg, lighter circles are lipid droplets, darker circles are *Symbiodinium* cells.

Parent colonies and gametes were sampled at three sites around Moku O Lo’e Island in Kane’ohe Bay, Hawai’i: Bridge to Nowhere (BTN), Gilligan’s Lagoon (GL), and Point Reef (PR) (Fig. 1) during the summers of 2007 and 2008. These sites are located at N 21° 25.893′ and W 157° 47.376′, N 21° 25.973′ and W 157° 47.392′, N 21° 25.988′ and W 157° 47.192′, respectively. *Montipora capitata* colonies exhibited primarily branching morphologies at BTN, plating at GL, and both branching or plating at PR (Fig. 2 a,b). The PR site was only sampled in 2008 and was included to examine whether *Symbiodinium* ITS2 sequence assemblages differed in corals sampled at a site where both branching and plating morphologies co-occur.

Egg-sperm bundles were released (spawned) between 8∶45 and 9∶15 pm during the first quarter of the new moon in June, July and August of 2007 and 2008. Samples of parent colonies at depths of 1–2 m were collected 5 d before spawning nights by removing small tissue fragments 4 cm away from tips and edges of the colony. Egg-sperm bundles released by corals at the BTN and GL sites were collected in the field using cylindrical nets placed over the adult colonies [41]. Due to rough conditions at PR in 2008, ten fragments of adult colonies were moved from PR into tanks (∼2 weeks before spawning) exposed to ambient light levels and gametes were collected as in [42]. After collection, egg-sperm bundles were broken apart and eggs were rinsed using 0.2 µm filtered seawater to remove potential external contaminants. Eggs from a given parental colony (Fig. 2 c & d) were combined and isolated together. All coral fragments and eggs were immediately frozen after collection and stored at −80°C until processed.

To compare the thermal environments at the collection sites, temperature was measured at 10 min intervals for 1 yr in 2007 using StowAway Tidbits accurate to ±0.2°C (Onset Computer) at BTN and GL. Light and temperature levels were also measured at all sites during two 2-wk periods in 2008 (22 September – 1 October and 25 November – 5 December, 2008). Light measurements were taken at 10 min intervals using Odyssey Photosynthetic Irradiance Recording Systems (Odyssey). These measurements were made to compare the light levels among sites and the timing of these analyses constrained by the availability of instrumentation. Temperatures were also recorded at PR during the same two 2-wk periods in 2008 (22 September – 1 October and 25 November – 5 December, 2008) that light levels were recorded. Both, temperature and light measurements were performed at ∼1.5 m depth, at the coral collection sites.

### Symbiodinium DNA Isolation, PCR, Cloning, Sequencing and Phylogenetic Analyses

Genomic DNAs from coral fragments and eggs were extracted using the guanidinium-based protocol described in [20]. The *Symbiodinium* ITS2 rDNA cistron was amplified (primers ‘ITS-DINO’ and ‘ITS2rev2’), cloned and sequenced following procedures detailed in [14]. DNA sequences were inspected and assembled using Sequencher v4.7 (Gene Codes Corporation, USA), identified via the Basic Local Alignment Search Tool (BLAST) in GenBank, and manually aligned with the BioEdit v5.0.9 sequence alignment software [43].

Sequences included in the downstream analyses met the following criteria: (1) they had either been published previously and the sequences retrieved and verified in multiple independent studies, or (2) were recovered in this study three or more times from clone libraries representing three or more independent coral samples. The remaining clone doubletons and singletons were excluded following [20]. Finally, ITS2 secondary structure folding was analyzed to identify potential pseudogenes as described in [37]. Two ITS2 alignments were then created for phylogenetic analyses, one for *Symbiodinium* clade C sequences and the other one for *Symbiodinium* clade D. Statistical parsimony haplotype networks were constructed using the software TCS version 1.21 [44] with a 95% connection limit and gaps were treated as a 5^th^ state.

The *Symbiodinium* ITS2 sequence assemblages or ‘*Symbiodinium* ITS2 assemblages’ herein, refer to the total number and taxonomic nature of different ITS2 sequences recovered from each sample analyzed. These *Symbiodinium* ITS2 assemblages were used as a proxy for *Symbiodinium* communities and to explore how *Symbiodinium* sequences partition among individuals and life stages. Sequence assemblages were obtained from 26 adult colonies of *Montipora capitata* and the eggs produced by each of these colonies (n = 52). Six of these adult colonies and their eggs (Colony ID: 8, 10, 19, 23, 29 and 37) were analyzed over two reproductive seasons (summers 2007 and 2008) to examine interannual variability in the *Symbiodinium* ITS2 assemblages (n = 12).

### Host DNA Isolation, PCR and Sequencing

A second subsample of the frozen adult corals was used to extract host DNA as described in [45]. The ATP synthetase subunit ß intron (*atpsß*) was amplified and sequenced as detailed in [37]. The resulting 300 bp sequences were edited and aligned using Sequencher v4.8 (Gene codes, Ann Arbor, MI). Gametic phases were determined computationally with Phase [46].

### Statistical Analyses

#### Symbiodinium ITS2 sequence assemblages

Data from sites BTN and GL (2007 and 2008) and site PR (2008) were analyzed separately because colony morphologies did not co-occur at all sites. Environmental and temporal effects on sequence assemblages in both parent and eggs were analyzed using data from BTN and GL sites. Morphological effects were analyzed using data from PR where both morphologies co-occurred. BTN and GL data were analyzed according to a four-factor experimental design (site, life-stage, year and colony). PR data was analyzed using a three factor experimental design (morphology, life-stage and colony). Analyses of molecular variance (AMOVA, [47]) were used to test whether the composition of sequence assemblages differed between factors (environments, years, life stages and morphology) using the genetic distance between the sequences as described in [37]. Matrices of simple pairwise genetic distances were generated in Arlequin v3.11 [48], the square root of each distance was taken, and the matrices were imported to Permanova+ v1.0.2 software add on for Primer 6 [49]. Due to the inability to differentiate inter and intragenomic ITS2 variants and resulting issues associated with the interpretation of ITS2 ‘type diversity’ as a proxy of ‘species delimitations’ [37], [50], which preclude us from unequivocally estimating *Symbiodinium* ‘type diversity’, this study used the ITS2 marker for a comparative analysis of sequence assemblage variation among samples. The goal of our study was to examine patterns of *Symbiodinium* ITS2 sequence assemblages between parent and egg coral samples, not to distinguish whether or how these sequences relate to the number of *Symbiodinium* ‘species’ present within a given sample. Thus, although ITS2 sequences cannot represent the “true diversity” of *Symbiodinium* [37], the ITS2 sequence assemblage approach is useful in distinguishing differences in patterns of *Symbiodinium* diversity among samples. As demonstrated in Stat et al. [37], if there is a significant difference in the ITS2 sequence composition detected by the AMOVA, this implies that *Symbiodinium* ITS2 assemblages are partitioned, regardless of the actual number of *Symbiodinium* types represented.

AMOVA, using simple pairwise genetic distance among alleles, was used to test for differences in genetic composition of different life stages for each colony according to [47]. Φ ranges between 0 to 1, Φ = 0 indicates identical genetic composition between samples, and Φ = 1 indicates alternate fixation of alleles. The percent similarity index (PSI, [51]) was estimated between adults and eggs for each coral colony; zero values of PSI index correspond to no similarity in the ITS2 sequence assemblages between life stages.

#### Host genetics

AMOVAs performed in Arlequin v3.11 [48] were used to test whether host genetic variation was partitioned by morphology or collection site. Global exact tests of non-differentiation [52] were then performed (α = 0.05, Markov chain steps = 10,000) to verify the results from the AMOVA.

#### Environment

Means, standard deviations and ranges (minimum–maximum) were calculated for each site during the periods sampled. A Kruskal-Wallis test was used to evaluate the differences in temperature and light between sites.

## Results

### *Symbiodinium* ITS2 Sequences in *Montipora Capitata*

**Figure 3:**
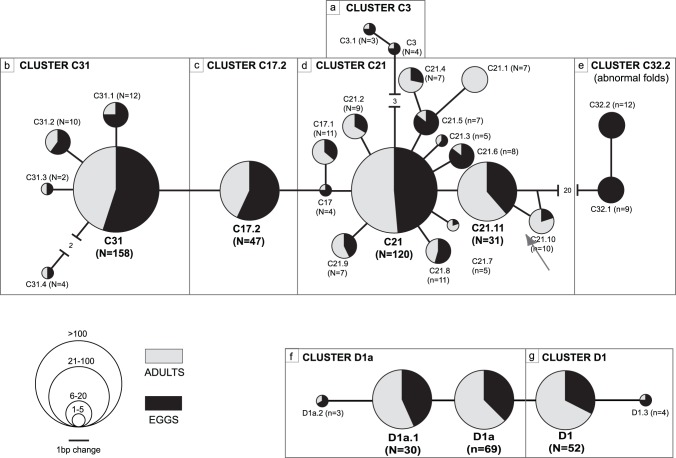
*Symbiodinium* sequence networks and folding clusters between parents and eggs. *Symbiodinium* ITS2 sequences (N = 659 sequences) identified from 64 *Montipora capitata* coral samples (see Table 1), showing the relationships among the 24 distinct ITS2 sequences retrieved in *Symbiodinium* clade C, and 5 in *Symbiodinium* clade D. The pie charts correspond to individual *Symbiodinium* ITS2 sequences, with the diameter of the pie charts proportional to the number of sequences retrieved corresponding to the circular inset scale (exact numbers given in brackets). Grey and/or black colors correspond to sequences obtained from adult coral colonies and coral eggs, respectively. Networks are subdivided into cluster groupings that each contains sequences with identical secondary structure folding. Details on secondary structures are shown in the Figure S1.

A total of 659 sequences were recovered from the 64 samples (32 adults and their respective eggs), representing 7–13 *Symbiodinium* ITS2 sequences per sample (10±1.87, average ± SE; Table 1, Fig. 3, GenBank accessions JF683321-JF683339). Our initial screen of sequences resolved 29 different ITS2 sequences that have either been published before, or were retrieved from multiple samples here. 24 of these sequences belonged to *Symbiodinium* clades C, and 5 to clade D (GenBank accession numbers in Table S1). Nine of the sequences matched previously published sequences (C3, C17, C21, C21.6, C21.11, C31, C31.1, D1, and D1a). The remaining 20 sequences were novel and were assigned names indicating the clade, the number of the most closely related published sequence type, and a decimal and a number to distinguish them from published types and one another [20].

**Table 1:** *Symbiodinium* ITS2 sequences in adults and eggs of *Montipora capitata*, and ATP synthetase subunit β genotypes for the adult corals sampled at three sites on Moku O Lo’e Island, Kane’ohe Bay, Hawai’i.

Site	Year	Colony ID	*Symbiodinium* ITS2 sequence/s ADULTS	*S*	*Symbiodinium* ITS2 sequences/s EGG	*S*	*PSI*	*Φ*	*p*	*G*
BTN	2007	1	C31^5^, C31.2^2^, C21^2^, C17^1^	1	C32.2^8^, C31^1^	1	0.50	0.807	<0.001*	U
		8	D1^4^, D1a^4^, D1a.1^2^	1	D1a^2^, C31^2^, C31.4^1^, C21.2^1^,C21.10^1^, C17.2^1^, C3^1^	2	0.22	0.734	<0.001*	H
		10	D1a^4^, D1^3^, D1a.1^2^	1	C32.1^6^, C31^2^, C17.2^1^	1	0.00	0.934	<0.001*	F
		11	D1a^7^, D1^1^, D1a.1^1^	1	D1a^5^, D1a.1^3^, D1^2^	1	0.72	−0.039	0.670	G
		12	D1^9^, D1a^4^	1	C31^4^, C17.2^2^, D1a^1^,C21^1^, C31.1^1^, C31.4^1^, C32.1^1^	2	0.10	0.875	<0.001*	I
		18	D1^3^, D1a^3^, D1a.1^2^, D1a.2^1^	1	C31^9^, C21^1^, C17.2^1^	1	0.00	0.987	<0.001*	M
		19	C31^3^, C21^3^, D1a^3^, D1^1^,D1a.1^1^	2	C31^5^, C21.11^2^, D1a^1^,C17.2^1^	2	0.38	0.157	0.072	N
	2008	8	D1^5^, D1a^4^	1	C21^3^, C31.1^2^, D1a^1^,C21.3^1^, C21.8^1^, C21.5^1^	2	0.11	0.856	<0.001*	H
		10	D1^5^, D1a^5^, D1a.1^1^	1	D1^4^, D1a.1^3^, D1a^2^, C21.11^1^	2	0.69	0.006	0.339	F
		19	C21^2^, C21.2^2^, C21.1^1^, C21.4^1^,C21.7^1^, C21.9^1^, C21.11^1^, C31^1^	1	C21^3^, C21.5^1^, C21.6^1^, C21.8^1^,C21.11^1^, C17.1^1^, C32.2^1^, D1a^1^	2	0.30	0.007	0.387	N
GL	2007	23	C17.2^5^, C31^3^, C17^1^, C21.11^1^ C3.1^1^	1	C17.2^3^, C31^3^,C17.1^1^, C21^1^, C32.2^1^	1	0.65	−0.045	0.912	T
		29	C31^8^, C21^1^	1	C31^5^, C31.1^1^, C31.2^1^,C3.1^1^	1	0.63	−0.055	0.527	D
		30	C21^4^, C31^3^,C17.2^2^, C21.11^2^	1	C31^3^, C17.2^3^, C17^1^, C21.3^1^, C31.1^1^, C31.3^1^	1	0.45	0.095	0.104	W
		35	D1a^6^, D1^3^, D1.3^1^,D1a.1^1^	1	D1a^5^, D1^4^, D1.3^1^	1	0.86	−0.074	0.811	E
		36	C31^5^, C21^2^,C17.1^1^, C31.4^1^, D1^1^	2	C31^7^, C32.1^2^,C31.1^1^	1	0.50	0.004	0.414	P
		37	C17.2^6^, C21.1^2^,C21^1^, C21.10^1^,C31.2^1^	1	C31^8^, C17.2^1^, C31.1^1^	1	0.10	0.500	<0.001*	E
		39	C31^6^, C31.1^2^,C17.1^1^, C21^1^, C31.3^1^	1	C31^6^, C31.2^2^, C31.1^1^, C31.3^1^ C3^1^	1	0.73	−0.028	0.718	G
	2008	23	C17.1^2^, C31^2^,C21^1^, C21.4^1^, C21.8^1^	1	C21^7^, C17^1^, C21.9^1^, C31.2^1^	1	0.14	0.044	0.241	T
		29	C21^7^, C31^2^	1	C21^9^, C31^1^	1	0.88	−0.057	0.578	D
		37	C21^5^, C21.10^3^, C21.2^1^, C21.11^1^,C31^1^	1	C31^3^,C21^2^, C21.8^2^, C21.3^1^, C21.5^1^, C21.11^1^	1	0.38	0.060	0.121	E
PR	2008	B1	C21^4^, C21.6^2^, C21.10^2^, C21.11^2^, C31.4^1^	1	C31^5^, C21^2^, C21.11^1^, C3.1^1^, D1^1^, D1a.2^1^	2	0.27	0.119	0.009	P
		B2	C21^4^, C31^4^, C21.11^2^, C17.2^1^	1	C17.2^4^, C21^2^, C21.4^1^, C21.6^1^,C21.7^1^, C21.11^1^, C31^1^	1	0.45	−0.011	0.403	D
		B5	C21^3^, C31^3^, C21.7^2^,C21.1^1^, C21.11^1^, C17.2^1^	1	C21^4^, C31^3^, C21.2^1^, C21.11^1^,C17.2^1^, D1^1^	2	0.73	−0.018	0.944	B
		B6	C21.9^3^, C31^2^, C21.4^2^, C17.1^1^, C21^1^, C21.10^1^	1	C31^5^, C17.2^3^, C21.5^1^, C17^1^,C32.2^1^	1	0.20	0.126	0.039	B
		B7	C21^4^, C31^3^, C17.1^1^, C17.2^1^, C21.6^1^, C21.11^1^	1	C31^3^, C31.1^2^, C31.2^2^, D1.3^2^, C21.11^1^,C32.2^1^	2	0.36	0.111	0.025	B
		B8	C31^5^, C21.8^2^, C21^1^, C21.1^1^, C21.11^1^, C31.1^1^	1	C21^6^, C17.2^2^, C31^2^,C21.5^1^, C21.9^1^,C21.10^1^	1	0.24	0.099	0.108	D
		P1	C31^4^, C21.1^2^, C21.2^2^, C17.1^1^, C21^1^, C21.11^1^	1	C21^4^, C21.6^2^, C31^2^, C17.2^2^,C21.5^1^	1	0.27	−0.023	0.515	J
		P2	C31^3^, C21^3^, C21.11^3^, C17.2^1^, C21.4^1^	1	C31^3^, C21^3^, C17.2^1^, C21.6^1^, D1a^1^	2	0.64	−0.003	0.430	L
		P5	C31^5^, C3^1^, C21^1^, C21.7^1^, C21.10^1^, C21.11^1^, C31.2^1^	1	D1a.1^5^, D1^4^, D1a^3^, D1a.2^1^	1	0.00	0.973	<0.001*	G
		P6	C31^3^, C21^3^, C21.3^2^, C21.11^2^, C21.2^1^	1	C21^5^, C31^3^, C21.4^1^, C21.11^1^, C3^1^	1	0.64	−0.055	0.839	B
		P7	D1a.1^7^, D1a^3^, C21^1^, C21.8^1^	2	D1a^4^, D1a.1^1^, D1^1^, C21^1^, C21.8^1^	2	0.54	−0.085	0.641	N
		P9	C21^6^, C21.5^1^, C21.8^1^, C31^1^, C17.2^1^	1	C21^4^, C21.11^2^, C21.2^1^, C21.3^1^,C21.8^1^, C31^1^, D1a.1^1^	2	0.55	−0.002	0.514	A

Site abbreviations: BTN – Bridge to Nowhere, GL – Gilligan’s Lagoon, PR – Point Reef. Column Headings: *S* - number of clades/sample, *PSI* – Percent similarity index (between life stages), *Φ* - genetic differentiation, G – adult genotype. Numerals superscripts indicate the number of times a specific *Symbiodinium* ITS2 sequence was retrieved.

**Figure 4:**
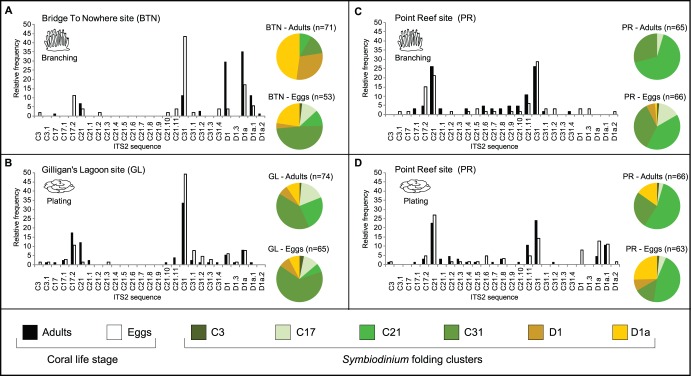
Abundance and distribution of *Symbiodinium* sequences and clusters between sites. The frequency of *Symbiodinium* ITS2 sequences per site (adult and egg) is displayed as bar graphs. The pie charts represent the frequency of *Symbiodinium* based on six of the seven ITS2 secondary structures (folds a, b, c, d, f and g; see Figure S1); note that fold type e did not meet our criteria for inclusion and was omitted from the downstream analysis.

The 29 ITS2 sequences grouped into seven secondary structural folds, 5 representing clade C sequences, and 2 clade D (Fig. 3, Fig. 4, Fig. S1). Less abundant sequences generally exhibited identical folding structures to the most closely related dominant sequence. Two sequences, C32.1 and C32.2, exhibited secondary structures that diverged significantly from the fold of their closest relative C21.11, and each resulted in abnormal folding conformation of helix IIIb (Fig. 3, Fig. S1). Based on these structural abnormalities, these sequences did not meet all our criteria for inclusion in the downstream statistical analyses and were excluded.

### Patterns in *Symbiodinium* ITS2 Sequence Assemblages

Individual coral and egg samples harboured from two to seven co-occurring ITS2 sequences (Table 1). The ITS2 sequences C31, C21, D1a, D1, C17.2, and C21.11 were common in parents and eggs being recovered 159, 119, 69, 52, 44, and 31 times respectively. The less abundant sequences generally differed from a dominant sequence by one to three base pairs. All ITS2 sequences were identified at least once in both life stages except C21.1, which was only detected in adults (Table 1, Fig. 3, Fig. 4). Similarly, all ITS2 sequences were detected at least once in both morphologies, except C31.3, which was only found in adults and eggs of plating morphologies from GL site (Table 1, Fig. 4). Fourteen of the 29 ITS2 sequences were found at all three sites (Table 1), however ITS2 sequences were not distributed evenly among sites with some being more abundant at one or two sites than the others (Table 1, Fig. 4).

We examined how ITS2 assemblages partitioned among colonies, life stages, sites, and time (Table 1). *Symbiodinium* ITS2 assemblages differed between BTN and GL sites (p = 0.0199) and between the adult colonies and their eggs (p = 0.0202, Table 2). There was also a significant interaction among all factors investigated (p = 0.0001, Table 2a), suggesting that *Symbiodinium* ITS2 assemblages can differ at the colony level (and within life stages) depending on the year and site. Φ statistics were higher in colonies from BTN (p = 0.005) than both other sites, reflecting larger differences in the assemblages between life stages at this site.

**Table 2:** Results of AMOVA testing for differences in *Symbiodinium* ITS2 assemblages among sites, years and life stage (BTN, GL sites) (A) and between morphology and life stage (PR site) (B).

	Test	Source	df	MS	Φ	P (perm)
A	Site, Years and Life stage	Si	1	2041.9	0.315	**0.0199**
		Ye	1	136.19	−0.035	0.4783
		Li	1	481.84	0.098	**0.0202**
		Co(Si)	12	269.98	0.025	0.4674
		SixYe	1	111.14	−0.118	0.5283
		SixLi	1	578.06	0.221	**0.0108**
		YexLi	1	59.716	0.013	0.2624
		YexCo(Si)	4	227.29	0.528	**0.0331**
		LixCo(Si)	12	56.832	0.241	0.2677
		SixYexLi	1	51.192	0.099	0.2581
		YexLixCo(Si)	4	29.479	0.390	**0.0001**
		Res	338	4.3134		
		Total	377			
B	Morphology andLife stage	Mo	1	307.67	0.076	0.1944
		Li	1	120.89	0.030	0.2185
		Co(Mo)	10	147.9	0.274	0.1281
		MoxLi	1	34.957	−0.036	0.4386
		LixCo(Mo)	10	58.791	0.453	**0.0001**
		Res	236	5.9302		
		Total	259			

Significance was determined by permutation test (10,000 permutations) of the pseudo-F statistic. Significant values (p<0.05) are indicated with bold font. Factor abbreviations: *Si* - site, *Li* – life stage, *Ye* - year, *Co* - colony, *Mo* – morphology, *Φ* - genetic differentiation.

Pairwise comparisons of sites, grouping ITS2 sequences by life stage indicated a significant difference in the *Symbiodinium* ITS2 assemblages in adult corals from BTN and GL (p = 0.0093), but not in their eggs (p = 0.2566). Although adult corals from BTN had a higher abundance of clade D sequences (ITS2 sequences D1, D1a, D1a.1) than those from GL, ITS2 sequence C31 was the most abundant in eggs from both sites (Table 1, Fig. 4a,b). In addition, pairwise comparisons of life stage within sites indicated a significant difference in *Symbiodinium* ITS2 assemblages between adult corals and their eggs from the BTN (p = 0.0408), but not at GL (p = 0.4186), with BTN adults having a greater proportion of clade D sequences than their eggs (Table 2, Fig. 4a). Despite the fact that eggs did not differ in *Symbiodinium* sequence composition between the two sites, we found significant differences in assemblages in eggs released by different colonies within each site. This indicates that there are major differences in the *Symbiodinium* ITS2 assemblages transmitted to eggs that are simultaneously released by adjacent coral colonies during a mass spawning event. Pairwise comparisons of life stage by colony in 2007 revealed that adult and eggs had different ITS2 assemblages in four of the seven colonies sampled at BTN (#8, 10, 12, 18, p = 0.0005, p = 0.0057, p = 0.0003, p = 0.0001 respectively), and in one out of seven colonies at GL (#37, p = 0.0004). No significant differences in the *Symbiodinium* ITS2 assemblages between adults and eggs were found when comparing samples from the same colonies taken in 2007 and 2008 (p = 0.4783, Table 2a). Generally, differences in the ITS2 assemblages between life stages were observed in parental colonies harboring predominantly clade D; their eggs contained higher abundances of clade C (Table 1). Percent similarity indices (PSI) showed a large range of values within each site (Table 1), highlighting the variability in ITS2 assemblages between life stages for each colony. *Montipora capitata* colonies at the BTN were predominantly branching, while those at GL were plating. To test whether the site differences in assemblages between BTN and GL were due to morphology, we analyzed corals sampled in 2008 from a third site (PR) where the two morphologies co-occur. This analysis revealed no significant difference in assemblages between plating and branching morphologies at this site (p = 0.1944, Table 2b), nor between life stages (p = 0.2185, Table 2b). The only differences in assemblages were between life stages and morphology within individual colonies, p = 0.0001 (Table 2b), a pattern that is consistent with the interpretation that individual colonies at this site had different assemblages of *Symbiodinium* ITS2.

The six ITS2 secondary structure folds were found in corals from all three sites. There were however, differences in the relative abundance of ITS2 folds at the three sites, which reflected the differences in the assemblages detailed above. For example, folding clusters D1 and D1a were most abundant at BTN site, whereas folding cluster C21 and cluster C31 were more abundant at the PR and GL sites, respectively (Fig. 4).

### Host Phylogenetic Analysis

A 280 bp fragment of *atpsß* was amplified from 48 adult colonies (20 from BTN, 15 from GL, and 13 from PR). The alignment identified 7 polymorphic sites and 17 distinct *atpsß* alleles among the 47 individuals. Using the Akaike information criterion (AIC) with a likelihood approach in Modeltest v3.06 [53], the best fit model of sequence evolution was HKY with base frequencies A = 0.3171, C = 0.1400, G = 0.1333, T = 0.4097 and a transition/transversion (Ti/Tv) value of 1.4826.

Branching and plating colonies of *Montipora capitata* shared common alleles supporting the hypothesis that the two morphologies are the same species. AMOVA results indicate that the majority of variance could be explained at the among individual level for groupings based on both morphology and collection site (Pvar = 49.09%; p<0.001 and Pvar = 52.80% p<0.001 respectively, Table 3). Variance at the highest hierarchical level was low and non-significant in both tests (p>0.05). Global exact tests of overall non-differentiation are significant indicating no partitioning based on differences between morphologies (p<0.0001) or collection site (p<0.0001). These results suggest a lack of genetic structuring due to either morphology or collection site at the scale examined in this study (Table 3).

**Table 3:** Results of AMOVA showing how genetic variance is partitioned for *M. capitata* when grouped according to morphology (A) and collection site (B) for the diploid nuclear locus atpsβ.

	Test	Source of Variation	df	SS	Variance Components	% of variation	Statistic	Value
A	Morphology	Among morphologies	1	13.339	0.16381	10.86	*Φ*_AM_	0.109
		Among samples within morphologies	2	8.576	0.12318	8.16	*Φ*_ AS(AM)_	0.092*
		Among individuals within samples	44	86.908	0.75322	49.92	*Φ*_AI(AS(AM)))_	0.616***
		Within individuals	48	22.500	0.46875	31.06	*Φ*_WI_	0.689***
		**Total**	95	131.323	1.50895			
B	Collection Site	Among sites	1	3.197	−0.12626	−8.96	*Φ*_ASI_	−0.090
		Among samples within sites	2	18.718	0.31282	22.21	*Φ*_AS(WSI)_	0.204**
		Among individuals within samples	44	86.908	0.75322	53.48	*Φ*_AI(WS)_	0.616***
		Within individuals	48	22.500	0.46875	33.280	*Φ*_WI_	0.667***
		**Total**	95	131.323	1.40853			

* *P*<0.05.

** P<0.005.

*** *P<*<0.001; statistical probabilities derived from 1023 permutations.

### Environmental Characteristics of Sites

Temperature differed at the three sites in both late summer and late autumn (H = 972.5, df = 2, p<0.0001, H = 69.2, df = 2, p<0.0001 respectively, Table S2). Temperature was higher and more variable at the BTN site, with up to ∼3°C fluctuations observed over a single 24-hr period (data not shown). Light levels differed significantly among the three collection sites (H = 57.9, df = 2, p<0.0001, H = 59.7, df = 2, p<0.0001, summer and autumn respectively). The BTN site had the broadest range of light levels in both summer and autumn sampling times (Table S2), and exhibited a recorded summer maximum of 1540 µmol quanta/m^2^s. The *Montipora capitata* colonies at this site were predominantly branching morphologies. The GL site had the lowest light levels where *M. capitata* were predominantly plating in morphology. Medium light levels were observed at the PR site where plating and branching morphologies of *M. capitata* co-occurred (Table S2). Overall, PR and GL sites had around 45% and 23% the light levels of BTN.

## Discussion

Parental effects in corals with vertical transmission of *Symbiodinium* have the potential to play a significant role in the phenotype of propagules, perpetuation of specific combinations of host-symbiont genotypes and ultimately the interaction of larvae with the environment. Vertical transmission increases the likelihood that offspring are seeded with *Symbiodinium* genotypes that are optimized to interact with the host. This strategy thus reduces the risk of forming unsuccessful symbiotic unions that might occur when acquiring *Symbiodinium* from the environment and that could reduce the growth and fitness of the coral [54]. This study is the first to explore *Symbiodinium* ITS2 assemblages vertically transmitted from parent to eggs in corals. Our results indicate that *Symbiodinium* ITS2 assemblages in the eggs of *Montipora capitata* are strongly influenced by the composition of the endosymbionts of the parent colony, and that the *Symbiodinium* ITS2 assemblages in the parent colonies differ and reflect characteristics of their physical environment.

A variety of *Symbiodinium* sequences were identified in the *M. capitata* adults and eggs, representing clades C and D, *Symbiodinium* lineages known to have different physiological characteristics and environmental thresholds. In this study, adults and eggs associated with clade D *Symbiodinium* were located in more challenging environments. For example, clade D *Symbiodinium* were found in branching colonies located in areas with high light and variable thermal regimes. This distribution is consistent with previous studies that document broader environmental thresholds for corals that associate with *Symbiodinium* clade D [22], [55]–[57].

Previous work on *Symbiodinium* diversity of *M. capitata* in O’ahu (Hawai’i) described a highly specific symbiosis between *M. capitata* brown morph and *Symbiodinium* ITS2 C31, and between the shallow orange morph and *Symbiodinium* ITS2 D1a [32]. In many cases the distinction between brown and orange morphs is ambiguous so we used shallow colonies that mostly resembled the “orange” morphotype. Our results revealed the presence of a much wider range of sequence types than previously reported in the “orange” morph, including the dominant types C3, C21, C31, D1 and/or D1a, and suggest that the presence and abundance of *Symbiodinium* ITS2 types are not specific to colony color or morphology. Despite the phenotypic plasticity of *M. capitata*, no host genetic differentiation was detected between sites or morphologies. This illustrates the important role that environment may play in structuring *Symbiodinium* ITS2 assemblages. For example, branching morphologies in a high light environment (BTN site) had higher abundances of ITS2 sequences D1 and D1a, whereas branching morphologies in a lower light environment (GL) had higher abundances of C31 and C21. Likewise, plate morphologies had C21 and C31 as dominant ITS2 sequences at PR and GL sites, respectively. Interestingly, plate morphologies at the PR site (medium light levels) showed higher abundances of ITS2 sequences D1 and D1a than plate morphologies at the GL site, which suggests that plate colonies may be experiencing a more challenging environment than branching colonies (which have more self-shading) at the PR site. *M. capitata* therefore appears to combine two strategies for acclimatizing to environmental change via differences in the composition of their *Symbiodinium* ITS2 assemblages and through its extraordinary morphological plasticity. Future experimental studies, using reciprocal transplants, should be conducted in order to fully validate the role played by the environment and the acclimatization strategies of the host.

The *Symbiodinium* ITS2 assemblages isolated from eggs were generally similar to their respective parent colony, encompassing anywhere from 2–7 *Symbiodinium* different sequences. It is unknown however, whether the nature of the *Symbiodinium* patterns in the eggs is controlled by the host or reflects competition within the *Symbiodinium* community and/or a race to occupy the less populated eggs [58]. To date, interactions between vertically transmitted symbionts remains underexplored and perhaps underestimated [59]. For example, it is unknown if a *Symbiodinium* type present in low abundance in an egg can proliferate and become dominant in the adult colony under the right environmental conditions, or if there is a threshold in abundance required for a *Symbiodinium* type to be viable in adult colonies.

The *Symbiodinium* ITS2 sequences in eggs from parents dominated by clade C were very similar in taxonomic composition to their parents. However, differences were detected in eggs originating from 4 of 7 parent colonies sampled at the BTN site, an environment where the corals exhibited branching morphologies. These 4 parent colonies all harbored clade D *Symbiodinium*; however, the eggs they produced all contained ITS2 assemblages with clade C and D. This result indicates that parent colony may preferentially transfer clade C *Symbiodinium* to their eggs rather than clade D. In coral species that are often dominated by *Symbiodinium* clade C versus D, clade D is often described as opportunistic [15], [60]. Although clade D has been shown to positively influence environmental thresholds in corals, there are known fitness tradeoffs, and corals hosting clade D do not grow as well as con-specifics that host clade C [24], [33], [55]. The idea that corals can detect these differences in physiology and preferentially select those that will provide the greatest benefit to their offspring is provocative and worthy of further investigation. Alternative explanations for these results include differences reflecting 1) environmental contamination of the eggs from free-living *Symbiodinium* cells, 2) sampling bias due to a single snapshot sampling of each adult coral colony investigated here, or 3) acquisition of *Symbiodinium* from the gastrovascular cavity environment rather than parental tissues.

The first scenario (environmental contamination) is less likely since a recent study shows that sequence for free-living *Symbiodinium* in sediments and seawater (near our study site) do not overlap with any endosymbiotic sequences obtained in *M. capitata* or other corals at the same study site [61]. Although it is possible that eggs could have also been infected by *Symbiodinium* cells recently released by other spawning colonies, we think that contamination to the egg would be minimal, since eggs are released as an egg-sperm bundle (surrounded by bundle material) and are quickly transported to the surface [62]. Furthermore, it is unclear if spawn-released *Symbiodinium* have the capacity to infect the eggs in the water column since transfer of *Symbiodinium* from the parents to the eggs is mediated by follicle cells present in the adult [58].

The second scenario (sampling bias) is more likely and is based on the fact that the eggs examined were released from multiple polyps located across the colony; in contrast, adult samples were taken from a single location on the colony. *M. capitata* colonies are extremely plastic in their colony morphology, and this structural complexity creates microenvironments with very different light regimes, micro-spatial variations that could influence the distribution of *Symbiodinium* within colony [63]. Indeed, spatial patterning of *Symbiodinium* clades as a result of differences in irradiance has been reported in *Montastraea* sp. [21] and *Acropora* sp. [64], [65]. It is also noteworthy that *M. capitata* has tissues that penetrate deeply into a porous skeleton. *M. capitata* eggs develop deep into the skeleton [62] and as such, acquire *Symbiodinium* from adult tissues within the skeleton that represent different microenvironments to surface tissues [66]. These differences may drive micro-zonation of *Symbiodinium* within coral polyps that create different likelihoods of infection depending on the closeness of the symbionts to the egg. Thus, the *Symbiodinium* ITS assemblages in the egg could reflect a combination of both parental selection of *Symbiodinium* and/or a stochastic infection depending on the *Symbiodinium* diversity present near the fecund polyps. Although such micro-spatial patterns of *Symbiodinium* are not well understood, they may have important ramifications for the performance of these corals and are worthy of further investigation. For example, future analysis on vertical transmission should compare how *Symbiodinium* diversity in individual eggs relates to the diversity in the polyp tissue and in the case of perforate corals, in the tissue located within the skeleton, in and around locations where the eggs develop [62].

Finally, the third scenario to consider, is that eggs of *M. capitata* acquire *Symbiodinium* cells transiently present in the gastrovascular cavity that have recently been expelled/acquired but that are not endosymbiotic with the host colony. This scenario is plausible since eggs are infected by *Symbiodinium* cells ∼2 weeks before release [38], [67], when some of the eggs are located along the mesenterial filaments near the mouth (Padilla-Gamino, personal observation). If that were the case, eggs could exhibit differences in their capacities to acquire symbionts “horizontally” from the gastrovascular cavity, opportunities that would reflect their relative location with respect to the mouth. Having the capacity to obtain symbionts transiently available in the gastrovascular cavity could be an important strategy for the transmission of *Symbiodinium* diversity in “vertically” transmitting coral-algal symbioses and requires further exploration.

Our data show that *M. capitata* colonies simultaneously release eggs during spawning events that overall contain very different *Symbiodinium* sequence assemblages and could confer different physiological attributes to larvae and/or juvenile corals. For example, Little et al. [24] found that juvenile *Acropora* (same family as *M. capitata*) grow faster when infected with clade C than with clade D, regardless of whether clade C was the homologous or heterologous subclade type. Furthermore, Abrego et al. [25] showed that *Acropora* juveniles infected with *Symbiodinium* type C1 had enhanced physiological tolerance (measured by photosynthesis, respiration and fluorescence) over juveniles infected with clade D. Juveniles with *Symbiodinium* type C1 also had higher ^14^C photosynthate incorporation and increased carbon delivery to the host [26]. The production of a pool of eggs containing individuals with *Symbiodinium* assemblages that exhibit different physiological optima could potentially allow larvae to exploit a variety of habitats and survive a range of environmental conditions both in the water column and after settlement. As such, this characteristic may serve as an adaptive strategy to maximize reproductive success when the environments that offspring face, vary unpredictably. Settling in environments similar to the parent may be more advantageous for the offspring if the early-stage acclimatization capabilities are limited (i.e. inability to change *Symbiodinium* assemblages and/or acquire new *Symbiodinium* from the environment, “switching/shuffling” [15], inability to change host morphology).

This study showed for the first time the *Symbiodinium* sequence assemblages in coral eggs derived from a vertical transmission system. Our results demonstrate that eggs feature a much wider range of sequence types than previously considered and that environment may play a significant role in parental effects of a coral with vertical transmission. Thus, *Symbiodinium* diversity in the eggs can be a dynamic trait under parental influence. The diverse array of early-stage holobionts highlights the fundamental importance of multidimensional specificity and flexibility in vertical transmission, which have the potential to significantly influence the biology and ecology of host and the *Symbiodinium* [21], [68], the evolutionary processes (i.e. speciation rates) and the perpetuation and evolution of coral holobiont mutualisms [69]. *Montipora capitata* is a coral with high morphological plasticity that is able to host multiple *Symbiodinium* genotypes, and these genotypes differ in abundance depending on the environment and the colony. By releasing eggs with different *Symbiodinium* compositions, *M. capitata* populations maximize the chances of the early-stage holobionts to recruit and grow in microenvironments with very different environmental conditions and possibly reduce competition between the recruits. The diverse array of early-stage holobionts could enhance the resilience of future generations of *M. capitata* and may possibly increase the potential for adaptive responses to rapid environmental change [70].

## Supporting Information

Figure S1 ***Symbiodinium*** **ITS2 secondary structures.** Distinct structure folds representing the 29 ITS2 sequences shown in Fig. 1 (schematized here on the upper left corner). Seven distinct fold clusters (a-g) were characterized based on criteria described in [37]. The seven secondary folding structures shown here correspond to the most dominant ITS2 sequence found in each cluster (i.e., C3, C31, C17.2, C21, C32.2, D1a, and D1). The location of mutations (insertions, deletions, or hemi-CBC changes) for each ITS2 sequence variant found in each cluster are indicated with a green arrow and corresponding variant number. Four sequence variants are not indicated here, because the observed mutations are found within the 5.8S rDNA (i.e., outside of ITS2 secondary structure). Furthermore, 1 out of 2 and 1 out of 3 observed mutations were also found within the 5.8S rDNA for sequences C21.4 and C21.1, respectively.
(TIF)

Table S1 **GenBank accession numbers for the** ***Symbiodinium*** **ITS2 sequences identified in the present study.**
(DOCX)

Table S2 **Temperature (°C) and light (µmol quanta/m^2^s) data from the three study sites in Moku O Lo’e Island, Kaneohe Bay Hawai’i.**
(DOCX)
