# Supplementary figures and images for: From Parent to Gamete: Vertical Transmission of Symbiodinium (Dinophyceae) ITS2 Sequence Assemblages in the Reef Building Coral Montipora capitata
Source: PLoS One. 2012 Jun 6;7(6):e38440. doi: 10.1371/journal.pone.0038440 (PMC3368852; doi:10.1371/journal.pone.0038440)

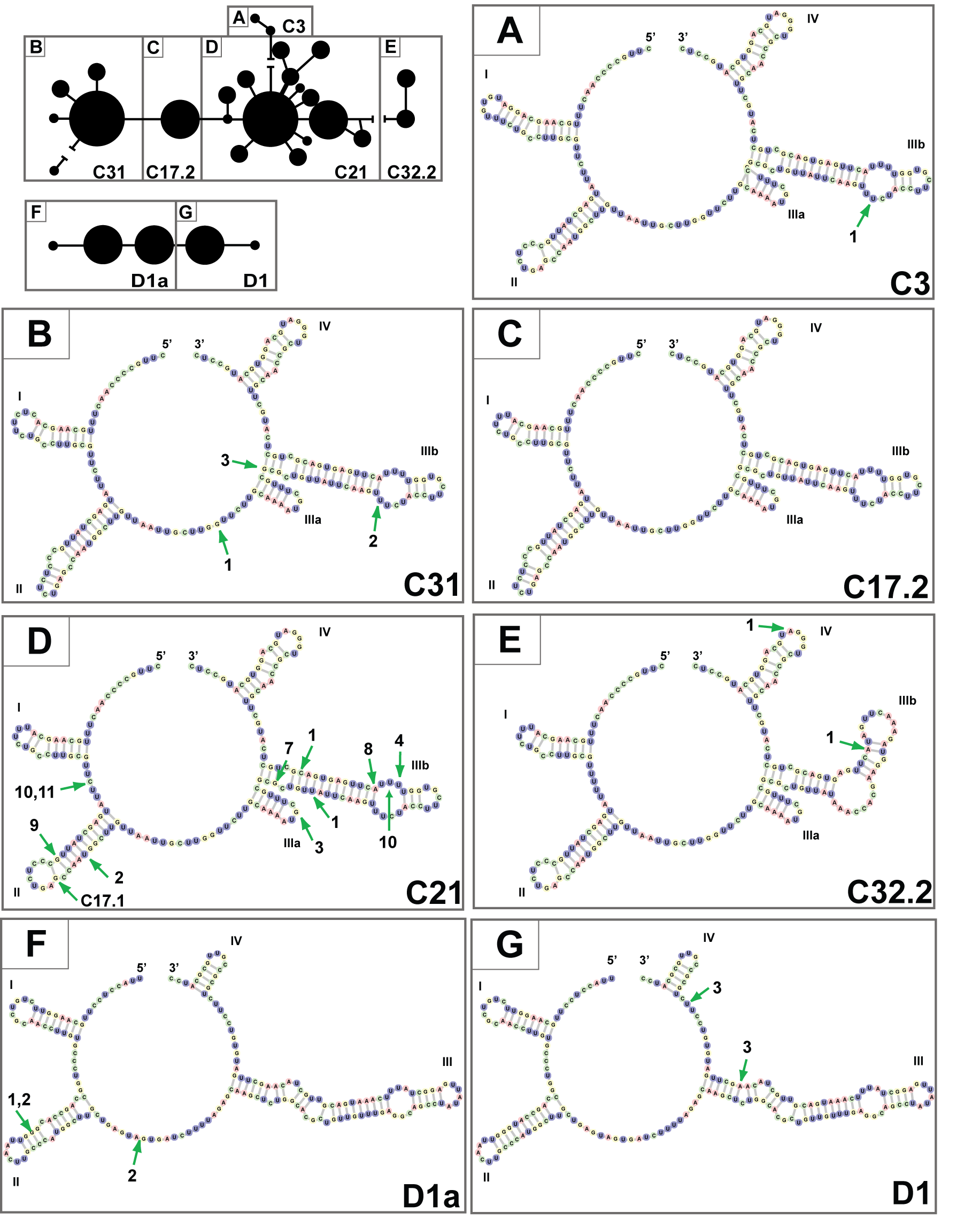

Supplement: Figure S1 — Symbiodinium ITS2 secondary structures. Distinct structure folds representing the 29 ITS2 sequences shown in Fig. 1 (schematized here on the upper left corner). Seven distinct fold clusters (a-g) were characterized based on criteria described in [37]. The seven secondary folding structures shown here correspond to the most dominant ITS2 sequence found in each cluster (i.e., C3, C31, C17.2, C21, C32.2, D1a, and D1). The location of mutations (insertions, deletions, or hemi-CBC changes) for each ITS2 sequence variant found in each cluster are indicated with a green arrow and corresponding variant number. Four sequence variants are not indicated here, because the observed mutations are found within the 5.8S rDNA (i.e., outside of ITS2 secondary structure). Furthermore, 1 out of 2 and 1 out of 3 observed mutations were also found within the 5.8S rDNA for sequences C21.4 and C21.1, respectively. (TIF) [file pone.0038440.s001.tif]
