# Supplementary material for: From Parent to Gamete: Vertical Transmission of Symbiodinium (Dinophyceae) ITS2 Sequence Assemblages in the Reef Building Coral Montipora capitata
Source: PLoS One. 2012 Jun 6;7(6):e38440. doi: 10.1371/journal.pone.0038440 (PMC3368852; doi:10.1371/journal.pone.0038440)
Supplement: Table S1 — GenBank accession numbers for the Symbiodinium ITS2 sequences identified in the present study. (DOCX) [file pone.0038440.s002.docx]

Supplementary Electronic Table 1. GenBank accession numbers for the *Symbiodinium* ITS2 sequences identified in the present study.

| ***Symbiodinium*** | |  |  |
| --- | --- | --- | --- |
| **Clade** | **Sub-clade sequence** | **GenBank Accession** | **Source** |
| C | C3 | AF499789 | LaJeunesse (2002) |
| C | C3.1 | JF683321 | Present study |
| C | C17 | AY239370 | LaJeunesse et al. (2003) |
| C | C17.1 | JF683322 | Present study |
| C | C17.2 | FJ461513 | Stat et al. (2009) |
| C | C21 | EU449102 | LaJeunesse et al. (2003) |
| C | C21.1 | JF683323 | Present study |
| C | C21.2 | JF683324 | Present study |
| C | C21.3 | JF683325 | Present study |
| C | C21.4 | JF683326 | Present study |
| C | C21.5 | JF683327 | Present study |
| C | C21.6 | HQ630873 | Stat et al. (2011) |
| C | C21.7 | JF683328 | Present study |
| C | C21.8 | JF683329 | Present study |
| C | C21.9 | JF683330 | Present study |
| C | C21.10 | JF683331 | Present study |
| C | C21.11 | HQ630874 | Stat et al. (2011) |
| C | C31 | AY258496 | LaJeunesse et al. (2004) |
| C | C31.1 | HQ630876 | Stat et al. (2011) |
| C | C31.2 | JF683332 | Present study |
| C | C31.3 | JF683333 | Present study |
| C | C31.4 | JF683334 | Present study |
| C | C32.1 | JF683335 | Present study |
| C | C32.2 | JF683336 | Present study |
| D | D1 | AF334660 | LaJeunesse (2001) |
| D | D1a | AF499802 | LaJeunesse (2002) |
| D | D1a.1 | JF683338 | Present study |
| D | D1a.2 | JF683339 | Present study |
| D | D1.3 | JF683337 | Present study |
|  | | | |
