# Supplementary material for: From Parent to Gamete: Vertical Transmission of Symbiodinium (Dinophyceae) ITS2 Sequence Assemblages in the Reef Building Coral Montipora capitata
Source: PLoS One. 2012 Jun 6;7(6):e38440. doi: 10.1371/journal.pone.0038440 (PMC3368852; doi:10.1371/journal.pone.0038440)
Supplement: Table S2 — Temperature (°C) and light (µmol quanta/m2s) data from the three study sites in Moku O Lo’e Island, Kaneohe Bay Hawai’i. (DOCX) [file pone.0038440.s003.docx]

Supplementary Electronic Table 2. Temperature (°C) and light (μmol quanta/m^2^s) data from the three study sites in Moku O Lo'e Island, Kaneohe Bay Hawai'i.

| **BRIDGE TO NOWHERE** | | |  |  |  |
| --- | --- | --- | --- | --- | --- |
|  | **Month** | **Mean** | **Max** | **Min** | **STD** |
| **Temp.** | Jul-07 | 27.5 | 30.6 | 26.0 | 0.8 |
|  | Aug-07 | 27.6 | 30.7 | 25.6 | 1.0 |
|  | Sep-07 | 27.7 | 30.8 | 24.4 | 1.0 |
|  | Oct-07 | 26.8 | 29.5 | 24.9 | 0.9 |
|  | Nov-07 | 26.0 | 29.2 | 24.2 | 0.9 |
|  | Dec-07 | 24.4 | 27.0 | 22.7 | 0.8 |
|  | Jan-08 | 23.6 | 26.5 | 21.5 | 1.0 |
|  | Feb-08 | 24.4 | 27.6 | 21.9 | 1.3 |
|  | Mar-08 | 25.7 | 28.0 | 24.1 | 0.8 |
|  | Apr-08 | 25.6 | 29.8 | 23.5 | 1.1 |
|  | May-08 | 26.9 | 30.7 | 24.6 | 1.2 |
|  | Jun-08 | 27.4 | 30.2 | 25.7 | 0.8 |
|  | Jul-08 | 28.6 | 31.2 | 26.8 | 0.9 |
|  | Aug-08 | 27.4 | 29.7 | 26.3 | 0.6 |
| **Temp.*** | Sep-08 | 27.4 | 29.3 | 22.7 | 0.6 |
|  | Nov-08 | 24.6 | 27.1 | 23.7 | 0.7 |
| **Light *** | Sep-08 | 245.3 | 1540.8 | 0.0 | 363.7 |
|  | Nov-08 | 161.2 | 1004.8 | 0.0 | 247.7 |
|  |  |  |  |  |  |
| **GILLIGAN'S LAGOON** | | |  |  |  |
|  | **Month** | **Mean** | **Max** | **Min** | **STD** |
| **Temp.** | Jul-07 | 27.3 | 29.0 | 26.4 | 0.4 |
|  | Aug-07 | 27.5 | 29.4 | 26.1 | 0.6 |
|  | Sep-07 | 27.3 | 29.2 | 24.4 | 0.7 |
|  | Oct-07 | 26.2 | 27.4 | 25.0 | 0.5 |
|  | Nov-07 | 25.4 | 26.9 | 24.0 | 0.6 |
|  | Dec-07 | 24.0 | 25.1 | 22.5 | 0.6 |
|  | Jan-08 | 23.0 | 24.6 | 21.4 | 0.7 |
|  | Feb-08 | 23.9 | 26.1 | 21.6 | 1.1 |
|  | Mar-08 | 25.3 | 27.1 | 24.2 | 0.6 |
|  | Apr-08 | 25.0 | 27.6 | 23.3 | 0.8 |
|  | May-08 | 26.4 | 28.8 | 24.7 | 1.0 |
|  | Jun-08 | 26.9 | 29.0 | 25.5 | 0.6 |
|  | Jul-08 | 28.1 | 29.9 | 26.7 | 0.6 |
|  | Aug-08 | 27.3 | 28.7 | 26.6 | 0.4 |
| **Temp.*** | Sep-08 | 27.3 | 28.5 | 22.4 | 0.5 |
|  | Nov-08 | 24.4 | 25.2 | 23.6 | 0.3 |
| **Light *** | Sep-08 | 57.6 | 582.1 | 0.0 | 92.1 |
|  | Nov-08 | 35.5 | 449.8 | 0.0 | 57.4 |
|  |  |  |  |  |  |
| **POINT REEF** | |  |  |  |  |
|  | **Month** | **Mean** | **Max** | **Min** | **STD** |
| **Temp. *** | Sep-08 | 27.0 | 29.1 | 23.1 | 1.6 |
|  | Nov-08 | 24.4 | 25.3 | 23.7 | 1.1 |
| **Light *** | Sep-08 | 124.8 | 838.5 | 0.0 | 192.7 |
|  | Nov-08 | 68.7 | 591.3 | 0.0 | 111.7 |

Asterisk (*) indicates that the sampling period was restricted to a short time interval (~2 weeks). Abbreviations: *max* – maximum, *min* - minimum, *std* - standard deviation, *cv* - coefficient of variation
